# Randomized trials of housing interventions to prevent malaria and Aedes-transmitted diseases: A systematic review and meta-analysis

**DOI:** 10.1371/journal.pone.0244284

**Published:** 2021-01-08

**Authors:** Kok Pim Kua, Shaun Wen Huey Lee

**Affiliations:** 1 Puchong Health Clinic, Petaling District Health Office, Ministry of Health Malaysia, Petaling, Malaysia; 2 School of Pharmacy, Monash University Malaysia, Sunway City, Malaysia; 3 Asian Center for Evidence Synthesis in Population, Implementation, and Clinical Outcomes (PICO), Health and Well-being Cluster, Global Asia in the 21st Century (GA21) Platform, Monash University Malaysia, Sunway City, Malaysia; 4 Gerontechnology Laboratory, Global Asia in the 21st Century (GA21) Platform, Monash University Malaysia, Sunway City, Malaysia; 5 Faculty of Health and Medical Sciences, Taylor's University, Subang Jaya, Malaysia; Mahidol-Oxford Tropical Medicine Research Unit, THAILAND

## Abstract

**Background:**

Mosquito-borne diseases remain a significant public health problem in tropical regions. Housing improvements such as screening of doors and windows may be effective in reducing disease transmission, but the impact remains unclear.

**Objectives:**

To examine whether housing interventions were effective in reducing mosquito densities in homes and the impact on the incidence of mosquito-borne diseases.

**Methods:**

In this systematic review and meta-analysis, we searched 16 online databases, including NIH PubMed, CINAHL Complete, LILACS, Ovid MEDLINE, and Cochrane Central Register of Controlled Trials for randomized trials published from database inception to June 30, 2020. The primary outcome was the incidence of any mosquito-borne diseases. Secondary outcomes encompassed entomological indicators of the disease transmission. *I*^2^ values were used to explore heterogeneity between studies. A random-effects meta-analysis was used to assess the primary and secondary outcomes, with sub-group analyses for type of interventions on home environment, study settings (rural, urban, or mixed), and overall house type (traditional or modern housing),

**Results:**

The literature search yielded 4,869 articles. After screening, 18 studies were included in the qualitative review, of which nine were included in the meta-analysis. The studies enrolled 7,200 households in Africa and South America, reporting on malaria or dengue only. The type of home environmental interventions included modification to ceilings and ribbons to close eaves, screening doors and windows with nets, insecticide-treated wall linings in homes, nettings over gables and eaves openings, mosquito trapping systems, metal-roofed houses with mosquito screening, gable windows and closed eaves, and prototype houses using southeast Asian designs. Pooled analysis depicted a lower risk of mosquito-borne diseases in the housing intervention group (OR = 0.68; 95% CI = 0.48 to 0.95; P = 0.03). Subgroup analysis depicted housing intervention reduced the risk of malaria in all settings (OR = 0.63; 95% CI = 0.39 to 1.01; P = 0.05). In urban environment, housing intervention was found to decrease the risk of both malaria and dengue infections (OR = 0.52; 95% CI = 0.27 to 0.99; P = 0.05).Meta-analysis of pooled odds ratio showed a significant benefit of improved housing in reducing indoor vector densities of both Aedes and Anopheles (OR = 0.35; 95% CI = 0.23 to 0.54; P<0.001).

**Conclusions:**

Housing intervention could reduce transmission of malaria and dengue among people living in the homes. Future research should evaluate the protective effect of specific house features and housing improvements associated with urban development.

## Introduction

Mosquito-borne diseases represent a major contributor to the burden of infectious disease globally, resulting in adverse financial, health, and societal impacts [1]. The incidence and geographical distribution of many mosquito-borne diseases are projected to grow with infections emerging in new areas or re-emerging in regions from which they have previously been eliminated [2–4]. Malaria and dengue are the most common mosquito-borne diseases in humans, with an estimated 212 million cases and 96 million cases reported respectively each year [5]. Annually, dengue illness costs approximately US$9 billion [6], whereas malaria spending is approximately US$5 billion [7, 8].

Insecticide-treated bednets and indoor residual spraying have been widely used to prevent the mosquito-borne disease transmission [9, 10]. However, the widespread insecticide resistance has significantly compromised the effectiveness of such interventions [11], suggesting a need for additional approaches. In tandem with the global call for action to make human settlements inclusive, safe, resilient, and sustainable by 2030 [12], large-scale investments can have a positive impact on various health and wellbeing outcomes, including but not limited to chronic respiratory tract diseases, mental health conditions, and infectious diseases such as enteric diseases, respiratory infections, malaria, and dengue fever [13, 14].

Historical records from Greek (circa 550 B.C.) and Roman era depicted large drainage schemes could reduce plague and fever. Other mechanical vector control methods such as sleeping in high buildings, use of bednets, and installation of bed curtains were noted in the 13th century [15]. The first malaria intervention of house screening was implemented among railway workers in Italy. Only 4% of those in the intervention group contracted malaria as compared to 92% in the non-intervened group [15, 16]. Since then, public health scientists have continued to reveal that simple changes in house design have the potential for protecting people against mosquito-borne diseases [16].

An existing systematic review evaluating the evidence for housing improvements to reduce malaria included 90 studies, of which only five were randomized trials [17]. A multi-country analysis of 29 survey data indicated that improved housing reduced malaria and had similar protective effect as insecticide-treated bednets [18]. More recently, a Cochrane review illuminated some evidence that screened houses may reduce malaria infection based on two published trials [19]. In view of randomized controlled trials remain the most robust method to provide reliable evidence on the real effect of an exposure or intervention [20], we performed a systematic review and meta-analysis to summarize the findings from randomized trials examining approaches related to both primary construction and modification of homes, and assessed the benefits of various housing interventions on prevention of mosquito-borne diseases.

## Methods

### Search strategy

We searched for articles indexed in 16 electronic databases, including PubMed, CINAHL Plus, LILACS, Ovid MEDLINE, Cochrane Central Register of Controlled Trials, Cochrane Database of Systematic Reviews, Cochrane Clinical Answers, ASCE Civil Engineering Database, ACP Journal Club, NHS Economic Evaluation Database, Allied and Complementary Medicine, Database of Abstracts of Reviews of Effects, Wiley Online Library, Emerald Insight, IEEE Xplore, and ICONDA®CIBlibrary Database from database inception to June 30, 2020. No search restrictions were applied on study population, setting, or language. Search strings included terms related to mosquito-borne diseases (“malaria” or “Plasmodium” or “dengue” or “Zika”), housing interventions (“house” or “building” or “window*” or “door”), and randomized trials (“randomised trial” or “randomized trial” or “randomly”). Further detailed search was specified in the S1 Appendix. The bibliographies of recent reviews and all relevant articles were scrutinized for additional studies.

### Study selection, inclusion, and exclusion criteria

Titles and abstracts were independently screened by two reviewers, followed by the retrieval and screening of full-text articles using the eligibility criteria described below. Any disagreements between the two reviewers were resolved by consensus and consultation of an external researcher whenever necessary.

Randomized trials of any mosquito-borne diseases were eligible for inclusion if they were published in English and evaluated one of the following type of housing interventions [21]:

**Table:** 

**Type of interventions**	**Examples**
**Primary construction**	
Construction materials	Wall, roof, door, and eave
Design	House built above ground level on stilts, or fewer or smaller windows
**Modifications to existing houses (Non‐insecticidal)**	
Screening	Covering of potential entry points (ceilings, eaves, doors, windows, or gable ends)
Eaves	Filling in of eaves
Wall maintenance	Filling in of cracks and crevices
**Modifications to existing houses (Insecticidal)**	
Eave tubes	Insecticide‐treated netting fitted into tubes inserted into closed eaves Eaves are closed and tubes with insecticide‐treated netting
Insecticidal screening	Screening potential entry points of house with insecticidal-treated netting

The numerous structural housing interventions could be divided into three categories:

1. Design and material specifications for primary construction
2. Modifications or additions to the physical structure of existing houses
3. Incorporation of non-insecticidal or insecticidal systems into existing house structures to reduce indoor mosquito densities

Studies employing insecticide-treated bednets or curtains as a single intervention were excluded because the evidence has been reviewed extensively and has long been recommended by the World Health Organization and the United States Centers for Disease Control and Prevention as a core intervention for the disease control [22–29]. Studies available only as an abstract (e.g., conference abstracts) or non-English research articles were also excluded.

### Data collection

Two reviewers independently extracted information from included studies using a standardized, predesigned table in Microsoft Word 2016, including (1) study population, number of participants enrolled, mean age, percentage of participants, and baseline clinical characteristics; (2) intervention details; (3) outcome measures; and (4) study results. Primary outcome of interest was the incidence of any mosquito-borne diseases defined as the occurrence of the infection cases in a study population for the whole study period. Secondary outcomes encompassed entomological indicators of the disease transmission, including indoor or outdoor mosquito density quantified by the numbers and characteristics of mosquitoes caught using techniques such as baits, light traps, knockdown catches, aspirators, or other methods.

### Risk of bias assessment

Two reviewers independently assessed the risk of bias of each included trial using RoB 2.0 [30]. Risk domains include randomization process, timing of identification, and recruitment of individual participants in relation to timing of randomization (cluster-randomized trials only), deviations from intended interventions, missing outcome data, measurement of outcomes, and selection of reported results. The overall risk of bias was classified as low if all domains were at low risk of bias, as high if at least one domain was at high risk of bias or multiple domains were judged to have some concerns, or as some concerns if at least one domain was judged to have some concerns. Any discrepancies between the authors were resolved through consensus.

### Statistical analysis

Narrative and tabular synthesis of data was performed for all included studies. For studies which were insufficient for a meta-analysis, the findings were only systematically reviewed. When the primary articles had adequate similarities in terms of study outcomes, a random-effects meta-analysis was carried out to generate a pooled odds ratio for mosquito vector densities and mosquito-borne diseases, specifically malaria and dengue. Stratified analyses were also conducted based on illness prevented (malaria or dengue), type of housing interventions (installation of mosquito traps—incorporation of systems into existing house structures; installation of screened doors and windows or installation of screened ceilings or full screening of doors, windows, or closed eaves—modifications or additions to the physical structure of existing houses), study settings (entirely rural, entirely urban, or mixed), and overall house type (traditional or modern housing).

We analyzed quantitative data using random-effects meta-analyses to generate a pooled odds ratio and 95% confidence interval for each dichotomous outcome or a weighted mean difference, and 95% confidence interval for each continuous outcome, if any. Forest plots were presented for each meta-analysis along with the *I*^2^ statistic which is used to quantify heterogeneity [31]. Funnel plots were checked for publication bias using Egger’s test for asymmetry [32]. We used the Grading of Recommendations Assessment, Development, and Evaluation (GRADE) system with GRADEpro GDT software to judge the quality of evidence of the meta-analyzed outcome [33]. Analyses were undertaken using RevMan for Windows (version 5.3) and Stata (version 14.0).

### Ethics statement

The study was a systematic review and did not require approval from an ethics committee.

## Results

**Fig 1:**
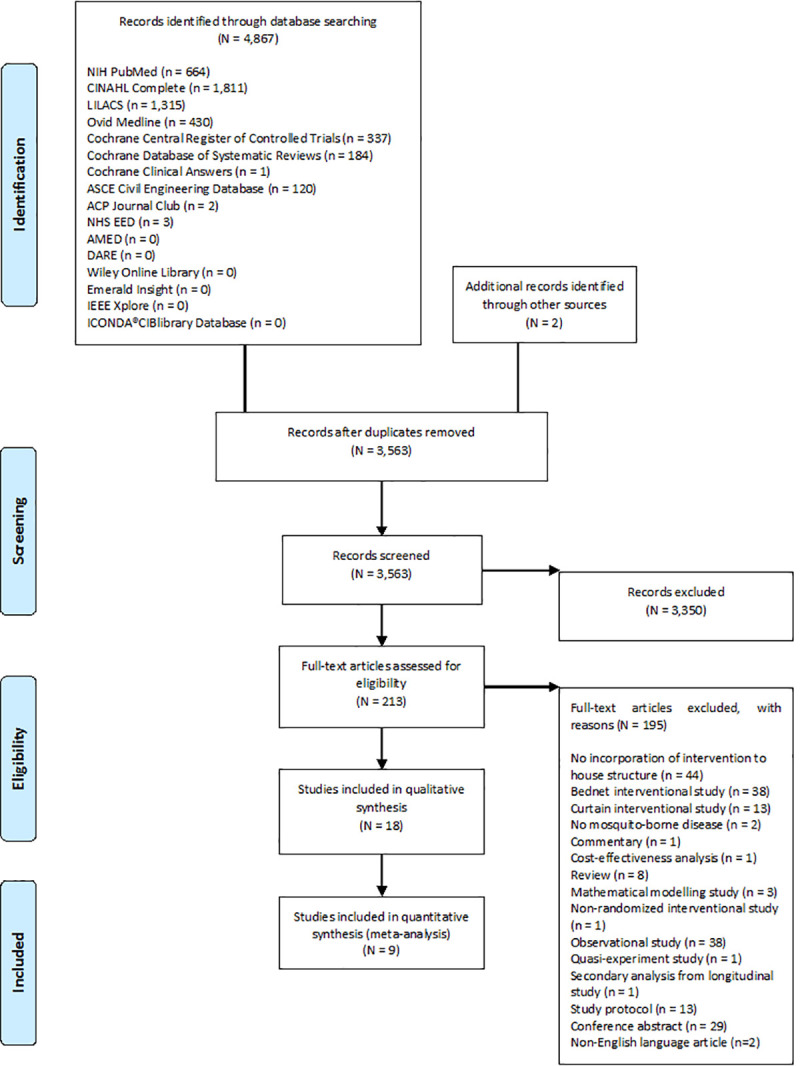
Flow diagram of study selection.

Of 4,869 studies identified, 18 were eligible for qualitative synthesis and nine for meta-analysis (Fig 1). Fourteen of the randomized trials were conducted in Africa [34–47], whilst four in South America [48–51]. The studies were published between 2003 and 2019, and enrolled approximately 7,200 households (Table 1). Four trials examined housing intervention on controlling Aedes and preventing the transmission of dengue, whereas fourteen trials evaluated on Anopheles and malaria transmission.

**Table 1:** Characteristics of included studies.

Study (year), country	Recruitment and baseline sample size	Intervention(s)	Control condition(s)	Duration of intervention	Outcome measures	Main findings
**Entomological (Aedes)**						
Che-Mendoza, et al. (2015), Mexico [48]	20 clusters of 100 households each	Duranet^®^ screens (0.55% w/w alpha-cypermethrin-treated non-flammable polyethylene netting) were mounted in aluminium frames custom-fitted to doors and windows of residential houses (n = 780 houses)	No intervention (n = 1,000 houses)	24 months	Indoor adult mosquitos collected using modified CDC backpack aspirators	At 5-month, significantly fewer houses of intervention group were infested with *Ae*. *aegypti* adult females (OR = 0.38; 95% CI = 0.21 to 0.69), blood-fed females (OR = 0.36; 95% CI = 0.21 to 0.60), and males (OR = 0.39; 95% CI = 0.19 to 0.77). Significant impact was still observed at 12-month post-intervention for adult females (OR = 0.41; 95% CI = 0.25 to 0.68) and males (OR = 0.41; 95% CI = 0.27 to 0.64).
Che-Mendoza, et al. (2018), Mexico [49]	20 clusters of 100 households each	Duranet® screens (0.55% w/w alpha-cypermethrin-treated non-flammable polyethylene netting) were mounted in aluminium frames custom-fitted to doors and windows of residential houses (n = 844 houses)	No intervention (n = 1,000 houses)	24 months	Indoor adult mosquitos collected using Prokopack aspirators	Significant reductions in the indoor presence and abundance of *Ae*. *aegypti* adults (OR = 0.48; IRR = 0.45; P<0.05 respectively) and *Ae*. *aegypti* female mosquitoes (OR = 0.47; IRR = 0.44; P<0.05 respectively) were observed in intervention group compared to control group.
**Entomological (Anopheles)**						
Atieli, et al. (2009), Western Kenya [34]	20 houses	Houses were modified with ceilings of papyrus mats to close eaves and small insecticide-treated nettings were incorporated in sleeping room ceilings and windows (n = 10 houses)	No intervention (n = 10 houses)	4 months	Indoor-resting mosquito densities determined based on pyrethrum spray collection method	84% *An*. *gambiae* reduction (OR = 0.16; 95% CI = 0.07 to 0.38) and 87% *An*. *funestus* reduction (OR = 0.13; 95% CI = 0.03 to 0.5) were observed in intervened houses compared to controls.
Jatta, et al. (2018), Gambia [47]	5 houses	(1) Modified modern house was built with mosquito screening and increased ventilation, including metal roof with ventilation in gables, closed eaves, complete mosquito screening, and well-fitted doors (n = 1 house)	Traditional house was built with thatched roof, open eaves, and poorly fitted doors (n = 1 house)	2 months	Indoor mosquitoes collected with a CDC light trap and mean indoor temperature for house	Closing the eaves of thatched houses resulted in 94% decrease in *An*. *gambiae* house entry (95% CI = 89 to 97) and increase in indoor temperature by 0.5°C (95% CI = 0.3 to 0.6) compared to thatched-roofed houses with open eaves. Metal-roofed houses with poorly fitted doors had three times more *An*. *gambiae* (Mean ratio = 2.99; 95% CI = 1.96 to 4.57) and were 1.5°C (95% CI = 1.3 to 1.7) hotter, and had 25% higher levels of carbon dioxide than thatched-roofed houses. In metal- roofed houses with closed eaves, mosquito numbers indoors were decreased by 96% with well-fitted screened doors. Improved ventilation with gable windows in metal-roofed houses made them as cool as thatched houses with open eaves.
		(2) Traditional house was built with metal roof, closed eaves, and poorly fitted doors (n = 1 house)				
		(3) Traditional house was built with thatched roof, closed eaves, complete mosquito screening, and well-fitted doors (n = 1 house)				
		(4) Traditional house was built with thatched roof, closed eaves, and poorly fitted doors (n = 1 house)				
Jawara, et al. (2018), Gambia [38]	37 houses	Screened doors and windows were constructed to prevent mosquito entry, provide security and privacy, and increase airflow, held in place with an aluminum frame (n = 24 houses)	No intervention (n = 6 houses)	10 weeks	Indoor mosquitoes sampled using light traps	All prototype doors and windows of intervention reduced the number of house-entering mosquitoes by 59 to 77% in comparison with the control houses (P<0.001).
Kampango, et al. (2013), Southern Mozambique [39]	16 houses	Netting materials (mosquito bednets, locally purchased untreated shade cloth or deltamethrin-impregnated shade cloth) against mosquito entry inside houses were applied over gables and eaves openings (n = 12 houses)	No intervention (n = 4 houses)	3 weeks	Mosquito entry rates assessed by light-trap collection	Entry rates of *An*. *funestus* were significantly reduced when the netting material was fitted over the gables of houses (IRR = 0.75; 95% CI = 0.62 to 0.91) and that extending the intervention over eaves did not enhance the protective effect (IRR = 0.80; 95% CI = 0.64 to 1.01). The netting materials significantly reduced entry of *An*. *gambiae* when applied over the gables (IRR = 0.17; 95% CI = 0.11 to 0.27) and both gables and eaves (IRR = 0.25; 95% CI = 0.17 to 0.37).
Kruger, et al. (2015), South Africa [46]	40 houses	(1) Western-style houses were built with brick and cement, and corrugated iron roofs or tiled roofs were incorporated with deltamethrin 0.52% w/w brown color lining, deltamethrin 0.85% w/w purple color lining, alpha-cypermethrin 0.29% w/w green color lining, or alpha-cypermethrin 0.47% w/w orange color lining (n = 16 houses)(2) Traditional mud huts were installed with deltamethrin 0.52% w/w brown color lining, deltamethrin 0.85% w/w purple colour lining, alpha-cypermethrin 0.29% w/w green color lining or alpha-cypermethrin 0.47% w/w orange color lining (n = 16 houses)	Western-style houses and traditional mud huts with no intervention on wall lining (n = 8 houses)	6 months	Knockdown and mortality rates of mosquitoes through WHO-recommended laboratory-scale contact or cylinder test and questionnaire-based data collection that included observations on the numbers of mosquitoes in the home	All four insecticide‑treated wall linings showed 100% knockdown and mortality of mosquitoes throughout 6-month post-installation in study homes. Thatch roofs and absence of ceiling in traditional mud huts increased mosquito access to dwellings. Gaps between roofs and tops of the walls (eave gaps) that were larger than 2 centimeters were present in 95% (19/20) of the traditional huts and 15% (3/20) of the modern houses, causing more mosquitoes in the homes. 65% (13/20) of participants in houses and 95% (19/20) in huts experienced irritation by mosquitoes while sleeping. Use of insecticides and repellents was higher among residents of huts.
Lindsay, et al. (2003), Gambia [41]	6 experimental huts (128 participants)	(1) Plywood ceiling (n = 1 hut)	No intervention (n = 1 hut)	6 weeks	Indoor mosquitos caught by traps	There were significantly fewer *An*. *gambiae* in huts with ceilings compared with controls: plywood (59% reduction), synthetic-netting (79%), insecticide-treated synthetic-netting (78%), plastic insect-screen (80%; P< 0.001 in all ceilings), and closed eaves (37%; P = 0.057). Likewise, netting and insect-screen ceilings reduced the number of *Mansonia spp*. by 70 to 72% (P<0.001) compared with controls.
		(2) Synthetic-netting ceiling (n = 1 hut)				
		(3) Insecticide-treated synthetic-netting ceiling (n = 1 hut)				
		(4) Plastic insect-screen ceiling (n = 1 hut)				
		(5) Eaves closed with mud blocks (n = 1 hut)				
		All ceilings were installed below the open eaves				
Massebo, et al. (2013), South-west Ethiopia [42]	40 houses	Doors and windows were screened by metal mesh and openings in the walls, and eaves were closed with mud. Any openings in the wall for ventilation purpose were closed by metal mesh only. Timber-framed was used for screening doors. Screened doors were fixed on the frame of the main door externally using hinges and were removed by rolling to enter or leave the houses. Windows were permanently fixed externally by metal mesh (n = 20 houses)	No intervention (n = 20 houses)	2 months	Indoor mosquitoes collected using CDC light trap	Mean number of *An*. *arabiensis* was 7.9 per light trap per night in control houses compared to 4.8 in houses with screened doors and windows, resulting in 40% fewer *An*. *arabiensis* in houses with intervention (P = 0.006).
Njie, et al. (2009), Gambia [43]	12 houses	Doors were screened and eaves were completely closed with a mixture of sand, rubble, and cement (n = 6 houses)	Screened doors with open eaves (n = 6 houses)	8 weeks	Indoor mosquitoes sampled using light traps	A 65% reduction in *An*. *gambiae* caught indoors (Mean number of *An*. *gambiae* per trap per night = 6.1 versus 2.1; OR = 0.34; 95% CI = 0.20 to 0.57) was reported in intervention group compared to controls.
Swai, et al. (2019), Southeastern Tanzania [44]	24 huts	Transfluthrin-treated eave ribbons were installed along eaves spaces (n = 12 huts)	Untreated eave ribbons (n = 12 huts)	7 weeks	Indoor and outdoor mosquito collections using light traps and carbon dioxide-baited BG® malaria traps	Intervention group showed decreased indoor densities of *An*. *arabiensis* by 77%, *An*. *funestus* by 60%, *Culex spp*. by 84%, and *Mansonia spp*. by 98% (P<0.001) compared to controls. Reductions in outdoor mosquito densities was also significant between intervention and control groups.
von Seidlein, et al. (2017), Northeastern Tanzania [45]	22 houses (40 participants)	Prototype houses of southeast Asian design were constructed with walls made of lightweight permeable materials (bamboo, shade net, or timber) with bedrooms elevated from the ground and with screened windows (n = 7 participants)	Modified and unmodified traditional African houses, wattle-daub or mud-block constructions, were built on the ground with poor ventilation (n = 33 participants)	9 months	Indoor mosquitos collected using Furvela tent traps during rainy season	There were fewer mosquitoes in prototype houses compared with traditional African houses, with double-storey houses showed the highest reduction in mosquito densities (96%; 95% CI = 92 to 98), followed by single-storey houses (77%; 95% CI = 72 to 82), and lowest in the modified reference houses (43%; 95% CI = 36 to 50) and traditional homes (23%; 95% CI = 18 to 29).
**Clinical (Aedes)**						
Degener, et al. (2014), Brazil [51]	1,487 households (6,300 participants)	BG-Sentinel® traps were installed in peridomestic area of houses such as verandas, kitchens, backyards, or indoors (n = 444 houses)	No intervention (n = 753 houses)	73 weeks	Mosquitos collected using BG-Sentinel® traps and cases of dengue virus IgM-seropositivity among residents	The intervention group had significantly less *Ae*. *aegypti* females captured during rainy seasons. The frequency of dengue virus IgM-seropositivity was marginally lower in intervention households compared with controls (Fisher’s exact test: P = 0.0624; OR = 4.97).
Degener, et al. (2015), Brazil [50]	775 houses	MosquiTRAP sticky traps were installed in peridomestic area of houses (n = 403 houses)	No intervention (n = 372 houses)	73 weeks	Mosquitos collected using BG-Sentinel® traps and cases of dengue virus IgM-seropositivity among residents	A higher abundance of female *Ae*. *aegypti* was collected in the intervention group (P = 0.008). There was no significant difference of mosquito abundance between intervention and control groups during the first rainy season (P = 0.141) and significantly higher abundance of female *Ae*. *aegypti* in the intervention arm during the dry season (P = 0.01) and second rainy season (P = 0.003). The frequency of dengue virus IgM-seropositivity was similar between houses in the intervention arm and the control arm (Fisher’s exact test: P = 1; OR = 0.59).
**Clinical (Anopheles)**						
Corbel, et al. (2012), West Africa [35]	28 villages (1,677 children)	Long-lasting insecticidal mosquito netting-universal coverage of sleeping units and full coverage of carbamate-treated plastic sheeting were lined up to the upper part of the household walls (n = 415 children)	(1) Long-lasting insecticidal mosquito netting-targeted coverage was given to pregnant women and children younger than 6 years (n = 429 children)	18 months	Incidence density rates of clinical malaria	There were no significant differences in incidence density of Plasmodium falciparum clinical malaria (Adjusted incidence density ratio = 1.05; 95% CI = 0.75 to 1.48), parasite densities of Plasmodium falciparum (Adjusted multiplicative coefficient = 0.98; 95% CI = 0.92 to 1.04) and prevalence of asymptomatic infections in children younger than 6 years (Adjusted OR = 0.81; 95% CI = 0.61 to 1.07) between the study groups.
			(2) Long-lasting insecticidal mosquito netting-universal coverage was given to all sleeping units (n = 413 children)			
			(3) Long-lasting insecticidal mosquito netting-targeted coverage was given to pregnant women and children younger than 6 years plus full coverage of carbamate- indoor residual spraying applied every 8 months (n = 420 children)			
Getawen, et al. (2018), South-western Ethiopia [36]	98 houses (477 participants)	Doors and windows of eligible houses were screened with wire-meshes. Screened doors were fixed on frame of main door externally using hinges. Windows screening was permanently fixed externally (n = 46 houses comprising 239 participants)	No intervention (n = 46 houses comprising 238 participants)	6 months	Mosquitos collected using CDC light traps and incidence of clinical malaria	There was an overall 48% reduction in indoor density of *An*. *arabiensis* (Mean ratio = 0.52) in intervention arm. The incidence of clinical malaria among residents of intervention group was significantly lower compared with control group (IRR = 0.39; 95% CI = 0.20 to 0.80).
Homan, et al. (2016), Western Kenya [37]	50 to 51 houses	Solar-powered odour-baited mosquito trapping systems were installed in households (n = 6,550 participants)	No intervention (n = 5,813 participants)	100 weeks	Incidence of clinical malaria and mosquito densities	Intervened clusters had 23 clinical malaria episodes, whereas non-intervened clusters had 33 episodes. Malaria prevalence measured by rapid diagnostic test was 29.8% (95% CI = 20.9 to 38.0) lower in clusters with intervention than in non-intervened clusters. The density of *An*. *funestus* was significantly lower in the intervention group compared to control group (Adjusted effectiveness = 69.2%; 95% CI = 29.1 to 87.4).
Kirby, et al. (2009), Gambia [40]	500 houses	(1) In homes with full screening, timber-framed doors and windows were constructed and covered with polyvinyl chloride-coated fibreglass netting (1.2-meter wide for doors, 2.4-meter wide for ceilings, and 1.0-meter wide for windows), with a mesh size of 42 holes per cm^2^. The gap between the top of the wall and roof (eaves) was filled with a mixture of sand, rubble, cement, and water (n = 200 houses)	No intervention (n = 100 houses)	12 months	Mosquitos collected using CDC light traps and incidence of malaria parasitemia	Mean number of *An*. *gambiae* caught in houses without screening was 37.5 per trap per night (95% CI = 31.6 to 43.3) versus 15.2 in houses with full screening (95% CI = 12.9 to 17.4) and 19.1 in houses with screened ceilings (95% CI = 16.1 to 22.1). Frequency of microscopically detectable malaria parasitemia was slightly higher in the control group than in either of the intervention groups (Full screening: OR = 0.79; 95% CI = 0.53 to 1.66; Screened ceilings: OR = 0.91; 95% CI = 0.54 to 1.70), although this was not statistically significant. There were no differences in the prevalence of high parasitemia (≥5000 parasites/μL): 6.3% in the control group, 4.2% in the full screening group, and 3.8% in the screened ceiling group.
		(2) In homes with screened ceilings, netting was stretched across the room below the eaves, fixed to the walls with wooden battens and any small holes were filled with mortar (n = 200 houses)				

### Methodological quality

11 out of 18 included studies (61.1%) were judged to have low risk of bias [34–37, 40, 43–45, 47–49]. Some concerns of risk of bias were identified with regard to randomization process in five randomized trials [38, 39, 41, 42, 46] and timing of recruitment of participants in two cluster randomized trials [50, 51] (S1 Fig). Due to the nature of the studies, it was not possible to blind the study participants and outcome assessors to intervention status.

### Housing interventions

Numerous housing modifications and designs were studied which included installation of ceilings to close eaves [41], closed eaves with modified ceiling and nettings in sleeping room ceilings and windows [34], screened doors and windows [36, 38, 42, 48, 49], nettings in sleeping room with carbamate-treated plastic sheeting lined up to walls [35], monofilament polyethylene insecticide-treated wall linings in traditional mud huts and modern type brick houses [46], nettings over gables and eaves openings [39], mosquito trapping systems in house [37, 50, 51], full screening of windows, doors, and closing eaves [40, 43], transfluthrin-treated eave ribbons to close eaves spaces [44], metal-roofed house with closed eaves, mosquito screening, and increased ventilation [47], and prototype houses of southeast Asian design built with walls made of lightweight permeable materials (bamboo, shade net, or timber) with bedrooms elevated from the ground and with screened windows [45].

#### Design and material specifications for primary construction

In one study, modern prototype houses of southeast Asian design were constructed. Single-storey houses were elevated on stilts whereas double-storey houses had upstairs bedrooms. The houses utilized permeable materials (cladding) for the construction of walls to maximize air ventilation and had a concrete or timber floor that was elevated from the ground for sleeping areas, a reinforced storage area that could be locked, an outdoor cooking area with chimney connected to the main building and covered by a roof, an outdoor latrine, and a water harvesting system that facilitated the collection of rain water through gutters and storage of water in a plastic container [45].

Another study evaluated a novel design of metal-roofed house that was screened and ventilated, consisting of screened and well-fitted doors, closed eaves, and triangular screened windows constructed with wooden frames and mosquito screening and were positioned in the gable ends of the building [47].

#### Modifications or additions to the physical structure of existing houses

The study by Njie and co-authors involved the installation of improved doors made of softwood, strengthened at the corners with polyvinyl chloride-coated fibreglass netting and had eave gaps closed thoroughly with a mixture of sand, rubble, and cement [43]. In the study by Massebo and co-workers, wooden framed doors and windows were screened by metal mesh, whereas openings on eaves and walls were closed with mud [42]. Similarly, another study fixed wire-mesh screening on the frame of main doors and windows externally [36]. Likewise, the study by Jawara and colleagues designed state-of-the-art screened doors and windows using a modular system, held in place using an aluminium frame [38]. In the research conducted by Kirby et al., houses with full screening were designed to have timber-framed doors and windows covered with polyvinylchloride-coated fibreglass netting, with gaps between the top of the wall and roof (eaves) filled with a mixture of sand, rubble, cement, and water [40].

In terms of insecticidal interventions, one study installed transfluthrin-treated eave ribbons along eaves spaces of houses [44]. Another study utilized modified ceilings such as plywood ceiling, synthetic-netting ceiling, deltamethrin-treated synthetic-netting ceiling, and plastic insect-screen ceiling for closing eaves [41]. Moreover, the study performed by Kampango and co-workers covered gables and eaves openings with bednets, untreated shade cloth, or deltamethrin-impregnated durable lining [39]. In two studies, the researchers mounted alpha-cypermethrin-treated non-flammable polyethylene netting in aluminium frames custom-fitted to doors and windows [48, 49]. The study by Atieli et al. modified houses with ceilings of papyrus mats to close eaves and fixed permethrin-impregnated netting in ceiling openings above sleeping room [34]. Another study utilized long-lasting insecticidal mosquito netting for coverage of sleeping units plus full coverage of carbamate-treated plastic sheeting lined up to the upper part of household walls [35]. The next study evaluated four insecticidal pyrethroid-impregnated polymer linings of different colors installed on inner walls of traditional mud huts and Western-style houses [46].

#### Incorporation of non-insecticidal or insecticidal systems into existing house structures to reduce indoor mosquito densities

Three studies have installed mass trapping systems in households. The study by Homan and colleagues incorporated a solar-powered odour-baited mosquito trap in each household. For two adjacent single-roomed households, one trap was being shared [37]. In the second study, three non-electrical sticky traps were installed in peridomestic areas (covered yard area, laundry area, or veranda) of each household, positioned at least five meters apart from one another [50]. The third study incorporated an electric-powered mass trapping system inside of home or in peridomestic areas such as veranda, kitchen, or backyard [51].

### Study findings

#### Entomological

Of 17 studies evaluating entomological outcomes [34, 36–51], 16 reported that housing intervention was effective in reducing the density of vector mosquitoes [34, 36–49, 51] (Table 1). One study utilized pyrethrum spray method that collected dead mosquitoes from a sheet and kept in a cooler box for laboratory quantification [34]. In two studies, mosquitoes were collected with aspirators for a 15-minute period per house [48, 49]. In addition, mosquitoes were caught using a light or carbon dioxide-baited trapping system in eleven studies [36–40, 42–44, 47, 50, 51]. A further study collected mosquitos using Furvela tent traps [45], whereas another study caught mosquitoes in the room and window traps [41]. Mosquitoes were also sampled using human landing catches technique in one study [35]. A study quantified residents’ perceptions based on observed number of dead mosquitoes on the floor, furniture, during cleaning, bites, and irritation, in addition to a laboratory-based analysis of knockdown and mortality rates of mosquitoes [46]. The most frequently caught mosquitoes in the studies were *Ae*. *aegypti* (dengue), *An*. *gambiae* (malaria), and *An*. *arabiensis* (malaria).

**Fig 2:**
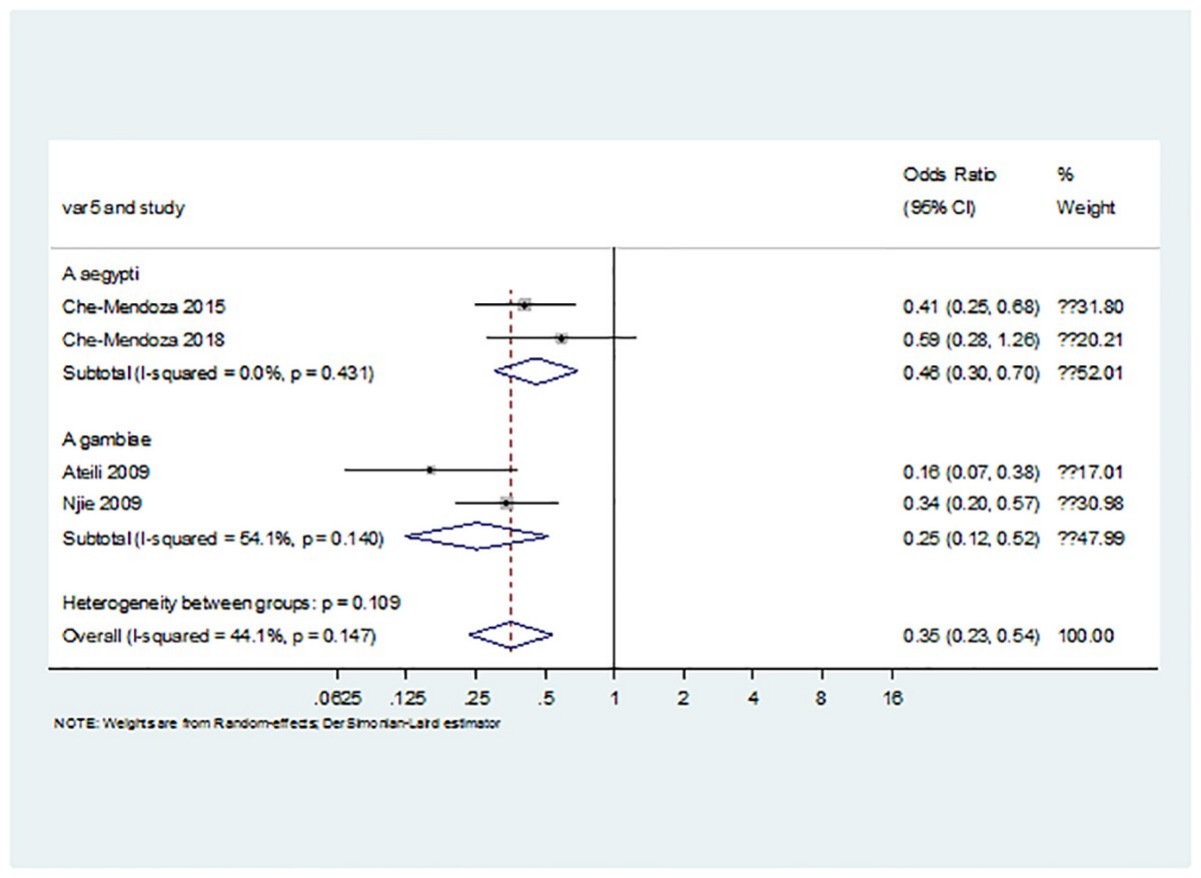
Pooled odds ratio for the effect of housing intervention in reducing mosquito vector densities.

In the pooled analysis of odds ratio reported in the primary studies, there was a significant benefit for improved housing on indoor vector densities of both Aedes and Anopheles (OR = 0.35; 95% CI = 0.23 to 0.54; P<0.001; Fig 2).

#### Clinical

Of three studies on malaria [35–37], two reported that housing intervention was effective in reducing the incidence (Table 1) [36, 37]. One study examined on parasitemia and found no evidence of an effect of housing intervention on the prevalence of malaria infection [40]. Two studies of dengue infection reported no statistically significant difference in the frequency of dengue virus IgM-seropositivity between houses in the intervention group than the control arm [50, 51].

**Fig 3:**
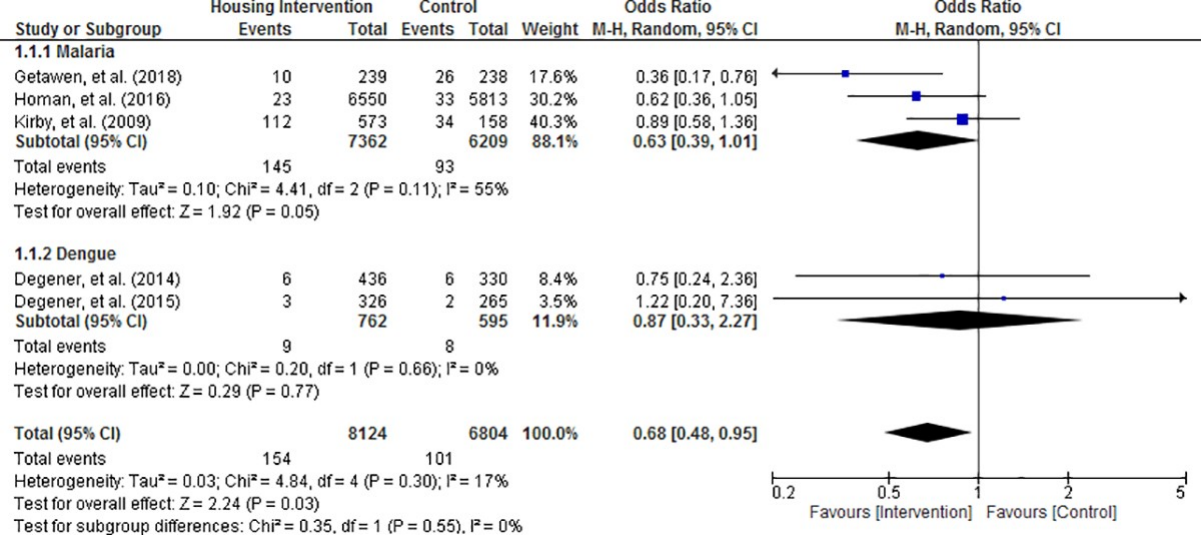
Effect of housing intervention on the risk of mosquito-borne diseases stratified by type of mosquito-borne diseases.

**Fig 4:**
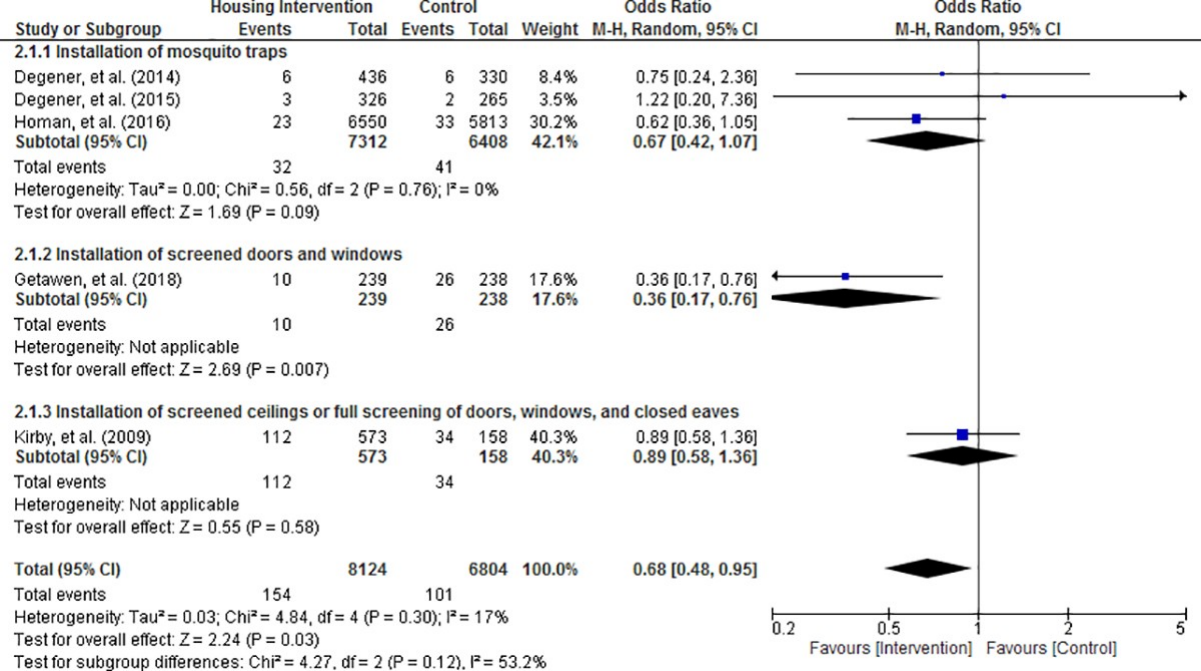
Effect of housing intervention on the risk of mosquito-borne diseases stratified by type of housing interventions.

**Fig 5:**
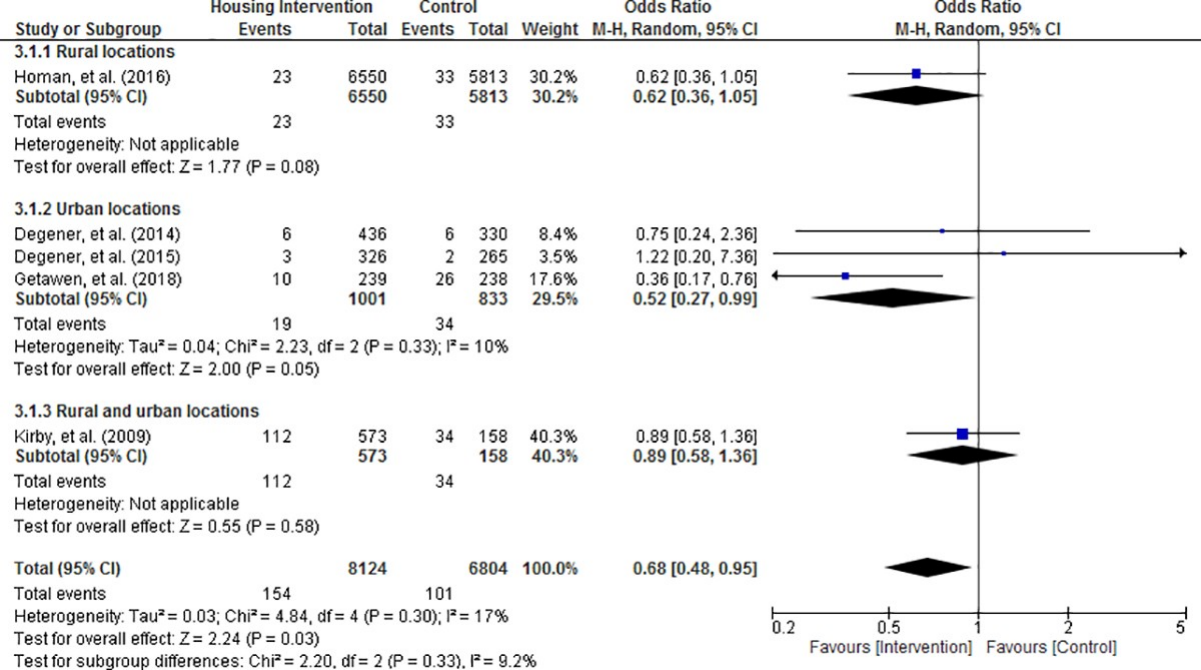
Effect of housing intervention on the risk of mosquito-borne diseases stratified by urbanicity.

**Fig 6:**
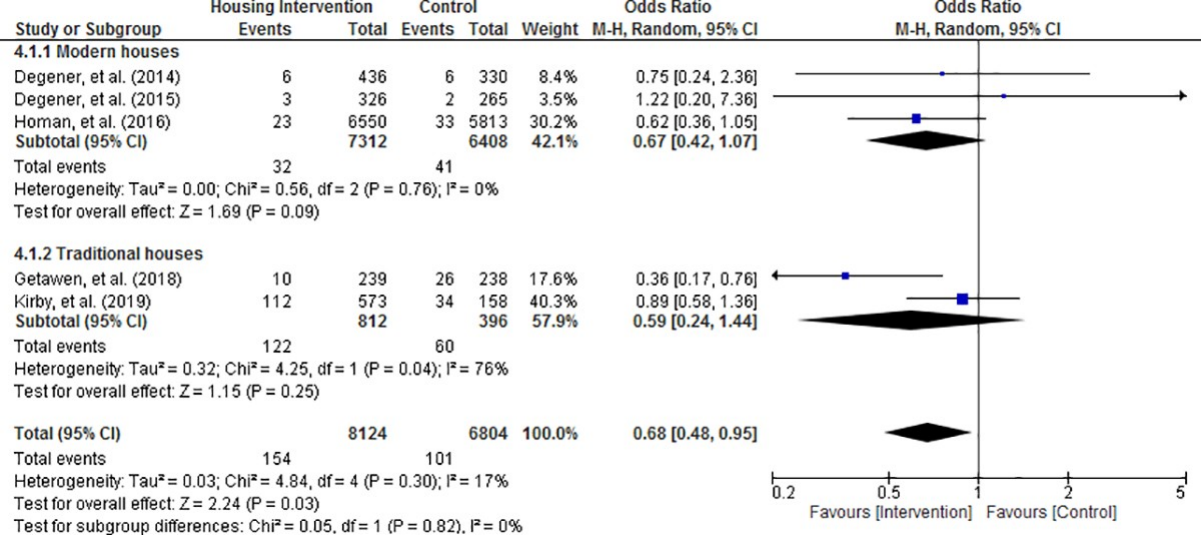
Effect of housing intervention on the risk of mosquito-borne diseases stratified by type of houses.

Random effects meta-analysis of the results revealed that the risk of acquiring mosquito-borne diseases was significantly reduced in housing intervention group compared with control condition (OR = 0.68; 95% CI = 0.48 to 0.95; P = 0.03; Figs 3–6). Visual inspection of the funnel plots did not show any sign of publication bias (S1 File). Subgroup analysis found that housing intervention had a significant benefit in reducing the risk of malaria in all settings (OR = 0.63; 95% CI = 0.39 to 1.01; P = 0.05; Fig 3) and the risk of both malaria and dengue in urban environment (OR = 0.52; 95% CI = 0.27 to 0.99; P = 0.05; Fig 5).

### Quality of evidence in meta-analysis

The certainty of retrieved evidence through GRADE assessments is presented in S1 Table. The incidence of mosquito borne diseases was rated as moderate, due to the serious imprecision from the wide confidence intervals. The certainty of evidence for the subgroup analyses varied; it was low for the incidence of malaria, moderate for incidence of dengue, and low to high with respect to incidence of mosquito-borne diseases under the subgroups of type of housing interventions, urbanicity, and overall house type.

### Community acceptability

In all eleven studies that evaluated community acceptance of housing improvements, positive responses were received. More than 90% of study participants cited satisfaction toward the installation of mosquito trapping system in houses and it was comfortable to use [50, 51]. Most community members believed that house screening improved privacy and prevented mosquitoes from entering [36, 38, 40]. Furthermore, modified ceilings were perceived to be essential in vector control and could improve the functionality and beauty of houses [34, 41]. Over 90% of study participants reported that they would use and pay for transfluthrin-treated eave ribbons installed along the eaves spaces as a means of mosquito prevention [44]. In the study by Kruger and colleagues, all of the study subjects indicated that they were pleased with the appearance, including color, position, and attachment method of the wall linings and agreed that the intervention resulted in decrease of indoor mosquitoes and other insects [46]. For prototype houses of southeast Asian design, residents expressed satisfaction with the new design, especially double-storey buildings because the bedrooms had more privacy, cooler indoor temperature, and were safer from insects and crawling animals. The community showed a preference for timber building material which they regarded as secure, durable, and protective of privacy [45]. In metal-roofed houses, ventilated houses were considered more comfortable compared to unventilated houses during the night when people retired to bed, nonetheless, those with closed eaves were deemed more uncomfortable than thatched-roofed houses because of the higher temperatures [47].

## Discussion

In this systematic review, we synthesized evidence from randomized studies conducted in malaria and dengue endemic tropical regions in Africa and South America, and depicted that housing intervention may offer protection against malaria and dengue. The results from our meta-analysis showed significant benefits overall for reducing the densities of the malaria and dengue vectors in homes, and reducing the incidence of clinical malaria but no significant effect on the incidence of dengue. Moreover, findings from individual studies reported that modified ceiling to close eaves, mosquito trapping systems, screened windows and doors, netting barriers to cover gable ends and eaves as well as prototype southeast Asian homes may reduce malaria or dengue transmission as depicted in the entomological outcomes.

Malaria and dengue are long-standing public health problems, particularly in the tropics. About a third of the world's population lives in regions where the climate is suitable for the transmission. To date, there is no effective dengue-specific prophylaxis or therapeutic. As such, an integrated vector management remains the only recommended approach for the disease prevention [52]. Space-spraying of insecticide to kill adult vectors in and around households is a popular approach, but, such method has not been eliciting a sustained positive impact on vector control [53]. It is noteworthy that malaria is mainly transmitted by mosquitoes indoor at night, hence, house modifications that decline mosquitoes from the indoor environment could lead to malaria incidence reduction. Dengue however is transmitted by the bite of the Aedes mosquito that typically attacks during daytime both inside and outside of the house. Therefore, the environment surrounding the house (waterbodies) rather than the house itself is likely to be the most important. It is very plausible and justifiable that malaria cases can be reduced by improved housing as indicated in our meta-analysis, nevertheless, dengue infections are more likely to be reduced by both housing and environmental management. Day-biting mosquitoes (Aedes), particularly females, are attracted to light during the day regardless of spectra. Their biting activity correlates with the time when people are also active outdoors, resulting in little protection against this mosquito species. Night-biting mosquitoes (Anopheles) specifically avoid ultraviolet and blue light during the day. Such behavioral attraction to and avoidance of light in both species change with time of day and show distinct sex and circadian neural circuit differences [54]. Genetic complexity and ecosystem diversity may cause behavioral changes and resistance in the mosquitoes, contributing to diminished effectiveness of insecticide-treated materials [55].

Modification of houses is a long-term, sustainable solution to control and eliminate of mosquito-borne diseases. Research suggest that this is achieved through two postulated mechanisms [17, 56]. Firstly, house entry by mosquito vectors is deterred by specific features in the homes such as closed eaves, the presence of ceilings, tiled, or metal roofs [18, 57]. These designs, which result in a higher daytime indoor temperature may impair mosquito survival and parasite development [58–61]. The architectural design of houses and choice of building materials equally play an important role. They may influence the existence of holes as routes of mosquito entry and the changes in indoor temperature and humidity [62]. In São Tomé of Africa, elevating house structure above the ground has been proven to reduce mosquito biting and indoor vector density [63]. More unscreened windows and open eaves or gables are likely to increase mosquito entry, hence, culminating in a higher risk of mosquito-borne infections [47]. While the design of openings in house structures is indispensable for ventilation and light, feasible intervention involves screening of the openings using appropriate building materials of which the effectiveness depends on the size and frequency of openings [38]. It is also possible to incorporate insecticides into housing materials albeit some concerns have been raised about photo-degradation of the quality upon prolonged exposure to sunlight as well as increase in the environmental levels of the chemical metabolites and their negative impact on the environment and the inhabitants of the house [64]. Our review identified a broad set of house improvements, consisting of chemical interventions (such as insecticides embedded in building materials) and physical interventions (such as screened doors and windows). Chemical interventions last much shorter than any physical interventions. Due to limited number of studies, our meta-analysis of clinical endpoint (incidence of illness) was able to pool data from studies of physical interventions. The pooled odds ratio of entomological endpoint showed a less pronounced effect associated with the two studies of chemical interventions [48, 49], demonstrating the interventions may be temporal compared to permanent (physical) interventions. Our review has indicated that eave ribbons can confer peridomestic protections against bites of malaria vectors. Such intervention is very useful for reducing the disease transmission risks in communities where people spend time outdoors during the day as well as early-night hours before going indoors or sleep under the bednets [65]. Likewise, mass trapping systems installed in peridomestic areas may have the benefit as well.

Our systematic review provides a timely contribution to the existing evidence base about the effects of housing interventions on all mosquito-borne diseases. To the best of our knowledge, it is the only review that focuses exclusively on randomized studies of malaria and dengue. The addition of this contemporary publication serves to address a paucity of such research design in housing interventions. Many of the included trials were conducted in some of the poorest communities, where often national health and development policies may not reach [66]. This can serve as a baseline for many countries in their future policy development planning to ensure that any public housing development can take these suggestions into consideration. Importantly, studies have shown that people with low socioeconomic status tend to spend more time at home [67–69]. This highlights the importance of housing improvements for the poor and socially disadvantaged groups who would be likely to spend more time at home. While housing quality is recognized as a prominent risk factor for a range of transmission settings [70, 71], our subgroup analysis could not find any discernible differences in effectiveness of interventions between residents of modern and traditional houses. A recent publication has similarly identified inconsistent correlation patterns between house type and prevalence of mosquito-borne disease within sub-Saharan African countries and suggested that it was caused by variations in the definitions of housing quality and conduct of surveys [18]. While a more pronounced advantage is observed in urban environments plausibly due to better access to health and social services, our data highlights the necessity of tailoring of the interventions for populations with different socioeconomic positions whose risk factor pattern and disease burden vary considerably.

Despite considerable variations in the complexity of housing interventions of the included studies, clinical and entomological evidence appeared to be consistently positive across the studies, thus highlighting the importance of comprehensive housing interventions as part of the epidemiological prevention of the infectious diseases. Our current study found that installation of screened doors and windows had a significant effect in reducing the risk of transmission of mosquito-borne diseases. Residents of both urban and rural settings would benefit from improved homes. Moreover, the potential health benefits of modern houses would go far beyond those built using traditional materials or designs. Further research is needed to investigate how different building elements contributed to clinically meaningful reduction in mosquito-borne illness. Reliance on a single intervention to control mosquito-borne diseases has often been ineffective, thus, systematic application of different interventions in combination and in synergy is anticipated to be a strategy with great promise [72]. While much remains a rather perplexing clinical puzzle, the effectiveness of existing multidisciplinary, comprehensive community-targeted intervention for various disease prevention would reasonably support the operation of future randomized clinical trials to evaluate housing as a strategic, long-term intervention for preventing mosquito-borne diseases. This is especially true taking into consideration that currently available or investigational malaria and dengue vaccines do not confer 100% rates of protective efficacy against the infections [73, 74]. At present, an increasing number of professionals and international organizations appreciate the strategies that feature “housing as a vaccine” to eliminate illness and disability. Stakeholders from both worlds of health and housing should be engaged in real-world case management, community-based counseling, and home-based health support services to ensure that the homes robustly meet the needs of people [75].

Our review findings possess global health implications. Mosquito-borne diseases will continue to exert healthcare and socioeconomic burden on numerous low- and middle-income countries across the world [76]. There is some evidence of the benefits of housing intervention on the prevention of mosquito-borne pathogens. However, the investments required for the construction of novel housing are markedly higher compared to indoor residual spraying and insecticide-treated bednets that cost less than USD$10 [77]. The health impact of novel designs in housing can go far beyond that to include decreased indoor mosquito density in tandem with a more comfortable environment for bednets to reduce mosquito-borne diseases, improved air quality, and availability of safe water and latrines to prevent other infectious diseases. The interventions might possibly translate into substantial improvements in morbidity, mortality and family health as well as social and economic impact attributable to the diseases. Government policies and current private housing expenditure determine the extent to which improved housing can be regarded as providing value for money. It is appropriate for government to promote public-private partnerships and deliver tax cuts to businesses that deliver healthy housing projects for community benefits [78]. Banks can offer microloan services for owners to make housing modifications. While the public sphere may exhibit a degree of scepticism with regard to provision of decent homes for political or humanitarian reasons, it is indeed crucial to collect more evidence along the lines of housing as a social determinant of health in the context of public expenditure. Integration of housing interventions within health and social care systems may improve physical, mental, and social wellbeing as well as reduce public health risks for infectious diseases and disability [79]. To glean a maximally precise picture concerning this nascent area of research, the approach would prove sufficient if it is bundled with clinical and cost-effectiveness findings from adequately powered, well-designed, and well-executed trials that can be applied to a diverse population, thereby garnering source of financial support from industry, public sector, and philanthropic organizations.

Several limitations of this review are worth noting. The sparse number of randomized trials published precluded our analysis for assessing a diverse range of building materials and architectural designs in the market. Most of the housing intervention research conducted thus far were observational studies [17]. It is interesting to note that evaluation of housing quality that was already present in the communities would yield a weaker evidence base compared to randomized trials that administered a direct intervention targeting communities who were known to be afflicted by mosquito-borne diseases. Overall, our findings broadly concurred with a recent Cochrane review that showed malaria infection may be reduced through improved house features [19]. Furthermore, majority of the included studies (61.1%) had comparatively short-term follow-up of 6 months or shorter. The effectiveness of such interventions on subjective outcomes such as quality of life, functional status, social or family wellbeing, and participants' satisfaction in housing conditions might require longer follow-up period to ascertain any differences and facilitate a thorough realist evaluation to determine what works, for whom and under what conditions [80, 81]. As such, it may present a spectrum of new challenges to be addressed in future research. While one of our included studies has involved migratory farmers, thus far, there have been very sparse number of community-level randomized controlled trials on mitigating mosquito-borne disease burden in humanitarian emergencies such as refugees, slums, and migratory communities, including fishermen, pastoralist, and forest workers who live in a poorly constructed house with no deployment of vector program. The unpredictable and volatile nature of these settings can often be restrictive pertaining to designing experimental studies [82]. In addition, all included studies were conducted in low- and middle-income countries. There has been a remarkable transformation of housing in urban and rural sub-Saharan Africa between 2000 and 2015, with the prevalence of improved housing has surged twofold from 11% to 23%, nonetheless, housing need is still acute given the rapid population growth [83]. Caution should be exercised in generalizing these interventions to other high-income countries with significantly different political, welfare, health, and socioeconomic systems. Effectiveness and cost-effectiveness of these interventions may vary across countries, nevertheless, further confirmation research is warranted in the settings based upon the concept of providing physical and chemical barriers to prevent mosquito entry.

Results of this review will hopefully encourage the development of mainstream policy discourse for the design of high-quality residential buildings to yield health benefits. It also resonates with the breath of interest of holistic sustainable development agenda to account for and remediate the incongruity of perpetuating substandard housing conditions and the attributable health inequities in resource-poor parts the world [14]. From a scientific, economic, and ethical perspective, appropriate housing interventions for implementation should be location and community-specific, effective, inclusive, acceptable, and affordable. Hence, the selection of the most appropriate housing interventions, combinations or enhanced design-led innovations must undergo initial pilot trials to provide a solid foundation for successive sizable scale-up of the project. Community-based housing interventional research that supports a collaboration between business, academia, and the public sector should be undertaken in multiple countries to give accurate, clear, location-specific, authoritative, scientifically sound, and economically viable policy recommendations.

## Conclusions

Housing intervention offers significant protection against malaria and dengue. Interventions such as screened doors and windows, improvements to roofs, ceilings, gables, eaves, or walls, mosquito trapping systems, and novel design houses hold promise for reducing dengue and malaria transmission.

## Supporting information

S1 Fig Risk of bias summary delineating authors' judgements about each risk of bias item for each included study. (DOCX)

S1 Table Certainty assessment of the included evidence via the GRADE approach. (DOCX)

S2 Table PRISMA 2009 checklist. (DOCX)

S1 File Funnel plots for meta-analyzed outcomes. (DOCX)

S1 Appendix (DOCX)
