# Supplementary material for: Randomized trials of housing interventions to prevent malaria and Aedes-transmitted diseases: A systematic review and meta-analysis
Source: PLoS One. 2021 Jan 8;16(1):e0244284. doi: 10.1371/journal.pone.0244284 (PMC7793286; doi:10.1371/journal.pone.0244284)

**S1 Fig. Risk of bias summary delineating authors' judgements about each risk of bias item for each included study.**


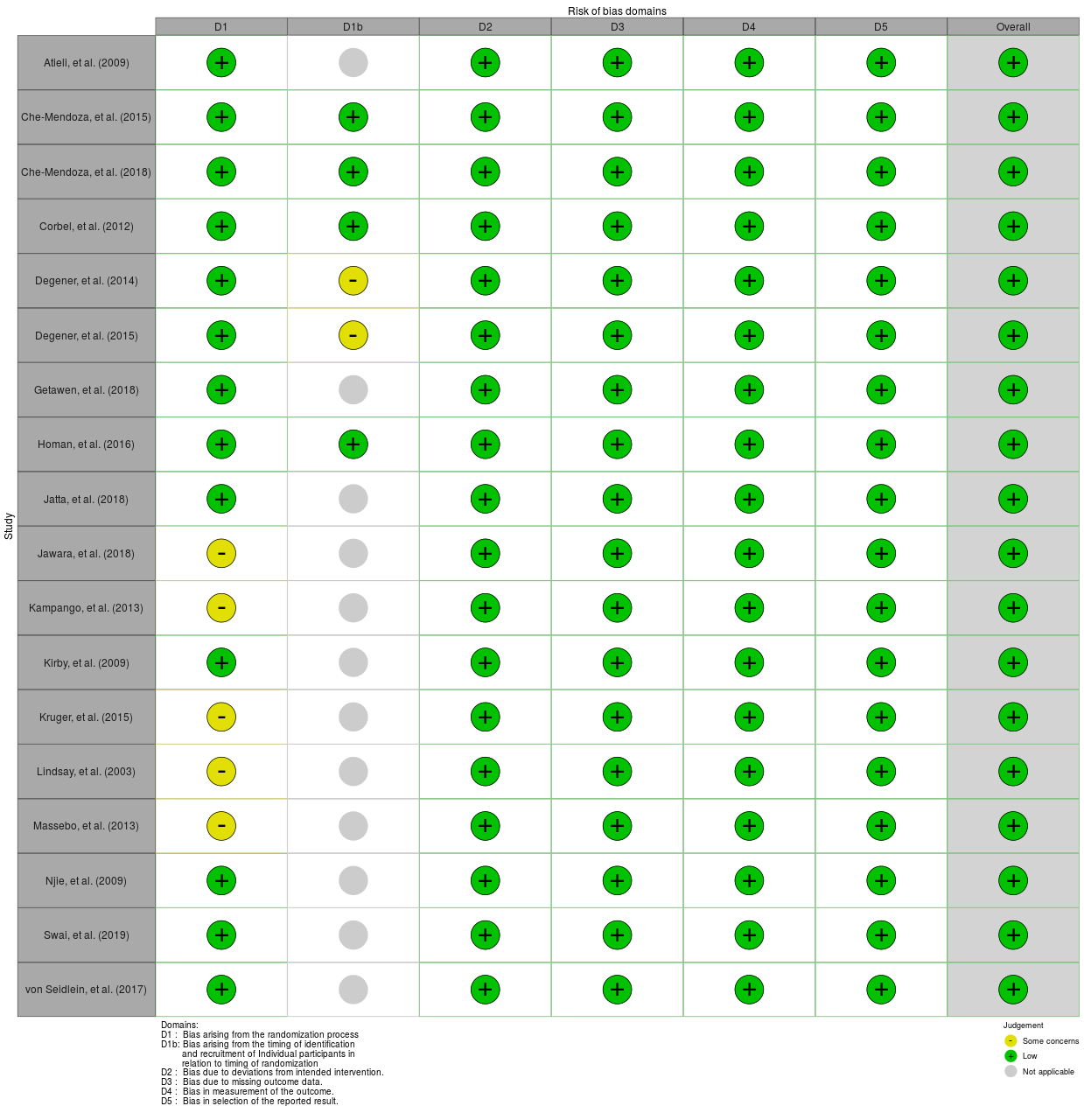

Supplement: S1 Fig — (DOCX) [file pone.0244284.s001.docx]
