# Supplementary material for: Randomized trials of housing interventions to prevent malaria and Aedes-transmitted diseases: A systematic review and meta-analysis
Source: PLoS One. 2021 Jan 8;16(1):e0244284. doi: 10.1371/journal.pone.0244284 (PMC7793286; doi:10.1371/journal.pone.0244284)
Supplement: S1 Table — (DOCX) [file pone.0244284.s002.docx]

**S1 Table. Certainty assessment of the included evidence via the GRADE approach.**

| **Certainty assessment** | | | | | | | **№ of patients** | | **Effect** | | **Certainty** | **Importance** |
| --- | --- | --- | --- | --- | --- | --- | --- | --- | --- | --- | --- | --- |
| **№ of studies** | **Study design** | **Risk of bias** | **Inconsistency** | **Indirectness** | **Imprecision** | **Other considerations** | **Housing intervention** | **Control** | **Relative**  **(95% CI)** | **Absolute**  **(95% CI)** |  |  |
| **Incidence of mosquito-borne diseases** | | | | | | | | | | | | |
| 5 | Randomized trials | Not serious | Not serious | Not serious | Serious | None | 154/8124 (1.9%) | 101/6804 (1.5%) | **OR 0.68** (0.48 to 0.95) | **5 fewer per 1,000** (from 8 fewer to 1 fewer) | ⨁⨁⨁◯ MODERATE | CRITICAL^a^ |
| **Incidence of malaria (subgroup analysis)** | | | | | | | | | | | | |
| 3 | Randomized trials | Not serious | Serious | Not serious | Serious | None | 145/7362 (2.0%) | 93/6209 (1.5%) | **OR 0.63**  (0.39 to 1.01) | **5 fewer per 1,000** (from 9 fewer to 0 fewer) | ⨁⨁◯◯  LOW | CRITICAL^b^ |
| **Incidence of dengue (subgroup analysis)** | | | | | | | | | | | | |
| 2 | Randomized trials | Not serious | Not serious | Not serious | Serious | None | 9/762 (1.2%) | 8/595 (1.3%) | **OR 0.87**  (0.33 to 2.27) | **2 fewer per 1,000**  (from 9 fewer to 17 more) | ⨁⨁⨁◯ MODERATE | CRITICAL^a^ |
| **Incidence of mosquito-borne diseases (subgroup of installation of mosquito traps)** | | | | | | | | | | | | |
| 3 | Randomized trials | Not serious | Not serious | Not serious | Serious | None | 32/7312 (0.4%) | 41/6408 (0.6%) | **OR 0.67** (0.42 to 1.07) | **2 fewer per 1,000**  (from 4 fewer to 0 fewer) | ⨁⨁⨁◯ MODERATE | CRITICAL^a^ |
| **Incidence of mosquito-borne diseases (subgroup of installation of screened doors and windows)** | | | | | | | | | | | | |
| 1 | Randomized trial | Not serious | Not serious | Not serious | Serious | None | 10/239 (4.2%) | 26/238 (10.9%) | **OR 0.36**  (0.17 to 0.76) | **67 fewer per 1,000**  (from 89 fewer to 24 fewer) | ⨁⨁⨁◯ MODERATE | CRITICAL^a^ |
| **Incidence of mosquito-borne diseases (subgroup of installation of screened ceilings or full screening of doors, windows, and closed eaves)** | | | | | | | | | | | | |
| 1 | Randomized trial | Not serious | Not serious | Not serious | Serious | None | 112/573 (19.5%) | 34/158 (21.5%) | **OR 0.89**  (0.58 to 1.36) | **19 fewer per 1,000**  (from 78 fewer to 56 more) | ⨁⨁⨁◯ MODERATE | CRITICAL^a^ |
| **Incidence of mosquito-borne diseases (subgroup of rural locations)** | | | | | | | | | | | | |
| 1 | Randomized trial | Not serious | Not serious | Not serious | Not serious | None | 23/6550 (0.4%) | 33/5813 (0.6%) | **OR 0.62**  (0.36 to 1.05) | **2 fewer per 1,000**  (from 4 fewer to 0 fewer) | ⨁⨁⨁⨁  HIGH | CRITICAL |
| **Incidence of mosquito-borne diseases (subgroup of urban locations)** | | | | | | | | | | | | |
| 3 | Randomized trials | Not serious | Not serious | Not serious | Serious | None | 19/1001 (1.9%) | 34/833 (4.1%) | **OR 0.52**  (0.27 to 0.99) | **19 fewer per 1,000**  (from 29 fewer to 0 fewer) | ⨁⨁⨁◯ MODERATE | CRITICAL^a^ |
| **Incidence of mosquito-borne diseases (subgroup of rural and urban locations)** | | | | | | | | | | | | |
| 1 | Randomized trial | Not serious | Not serious | Not serious | Serious | None | 112/573 (19.5%) | 34/158 (21.5%) | **OR 0.89**  (0.58 to 1.36) | **19 fewer per 1,000**  (from 78 fewer to 56 more) | ⨁⨁⨁◯ MODERATE | CRITICAL^a^ |
| **Incidence of mosquito-borne diseases (subgroup of modern houses)** | | | | | | | | | | | | |
| 3 | Randomized trials | Not serious | Not serious | Not serious | Serious | None | 32/7312 (0.4%) | 41/6408 (0.6%) | **OR 0.67** (0.42 to 1.07) | **2 fewer per 1,000**  (from 4 fewer to 0 fewer) | ⨁⨁⨁◯ MODERATE | CRITICAL^a^ |
| **Incidence of mosquito-borne diseases (subgroup of traditional houses)** | | | | | | | | | | | | |
| 2 | Randomized trials | Not serious | Serious | Not serious | Serious | None | 122/812 (15.0%) | 60/396 (15.2%) | **OR 0.59**  (0.24 to 1.44) | 56 fewer per 1,000  (from 110 fewer to 53 more) | ⨁⨁◯◯  LOW | CRITICAL^b^ |

^a^ Downgraded by 1 level for imprecision due to very wide confidence intervals.

^b^ Downgraded by 1 level for inconsistency because the heterogeneity between studies was substantial (*I*^2^=50% to 90%) and downgraded by 1 level for imprecision because the confidence intervals were wide.
