# Supplementary material for: Randomized trials of housing interventions to prevent malaria and Aedes-transmitted diseases: A systematic review and meta-analysis
Source: PLoS One. 2021 Jan 8;16(1):e0244284. doi: 10.1371/journal.pone.0244284 (PMC7793286; doi:10.1371/journal.pone.0244284)

Funnel plot for the effect of housing intervention on the risk of mosquito-borne diseases stratified by type of mosquito-borne diseases.


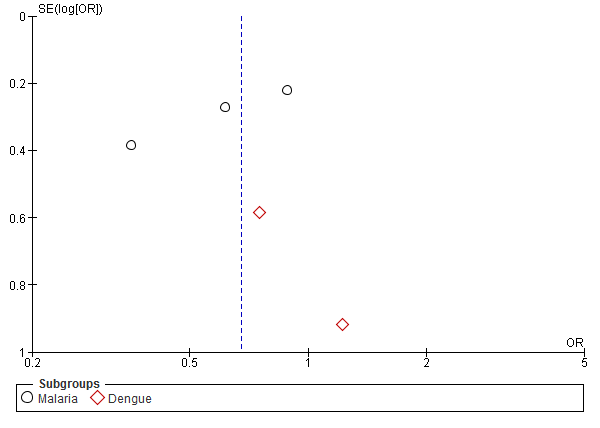


Funnel plot for the effect of housing intervention on the risk of mosquito-borne diseases stratified by type of housing interventions.


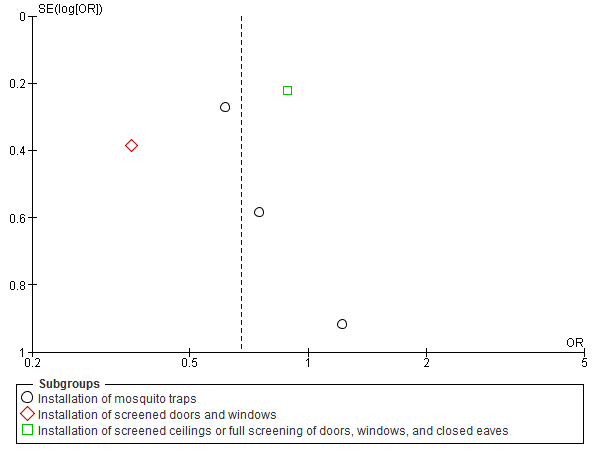


Funnel plot for the effect of housing intervention on the risk of mosquito-borne diseases stratified by urbanicity.


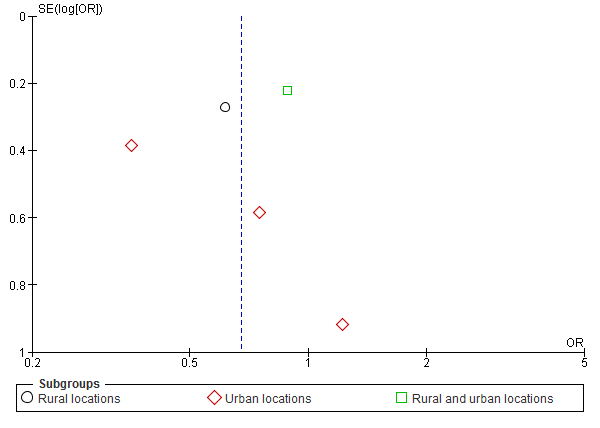


Funnel plot for the effect of housing intervention on the risk of mosquito-borne diseases stratified by type of houses.


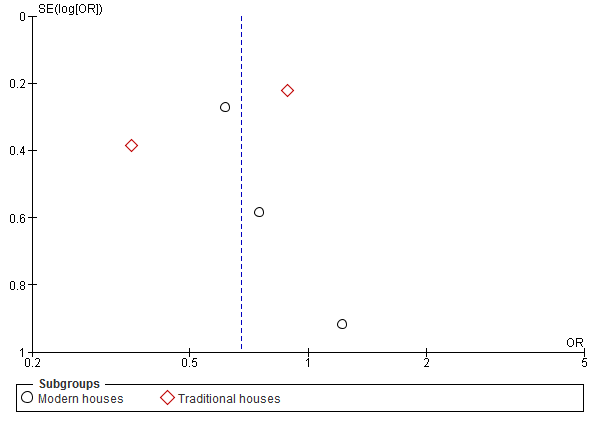

Supplement: S1 File — (DOCX) [file pone.0244284.s004.docx]
