# Supplementary material for: Randomized trials of housing interventions to prevent malaria and Aedes-transmitted diseases: A systematic review and meta-analysis
Source: PLoS One. 2021 Jan 8;16(1):e0244284. doi: 10.1371/journal.pone.0244284 (PMC7793286; doi:10.1371/journal.pone.0244284)
Supplement: S1 Appendix — (DOCX) [file pone.0244284.s005.docx]

**S1 Appendix**

**Search strategy**

malaria or Plasmodium or Anopheles or mosquito control or dengue or Aedes or chikungunya or Zika or yellow fever or West Nile virus or Eastern equine encephalitis or Japanese encephalitis or La Crosse encephalitis or St Louis encephalitis

AND

house or houses or housing or home or homes or hut or huts or building or buildings or dwelling or dwellings or shelter or shelters or architecture or roof or roofing or wall or walls or eave* or window* or door or doors or airbrick* or air brick* or ceiling* or stilt or stilts

AND

randomised trial or randomized trial or randomly
